# Axotomy-induced neurotrophic withdrawal causes the loss of phenotypic differentiation and downregulation of NGF signalling, but not death of septal cholinergic neurons

**DOI:** 10.1186/1750-1326-5-5

**Published:** 2010-01-19

**Authors:** Oscar M Lazo, Jocelyn C Mauna, Claudia A Pissani, Nibaldo C Inestrosa, Francisca C Bronfman

**Affiliations:** 1 Department of Physiology, Neurobiology Unit, Center of Ageing and Regeneration (CARE), Nucleus Millennium in Regenerative Biology (MINREB), Faculty of Biological Sciences, Pontificia Universidad Catolica de Chile, Alameda 340, CP 8331010, Santiago, Chile; 2 Department of Cellular Biology, Center of Ageing and Regeneration (CARE), Faculty of Biological Sciences, Pontificia Universidad Catolica de Chile, Alameda 340, CP 8331010, Santiago, Chile; 3 Current address: Department of Neurobiology, University of Pittsburgh, Pittsburgh, PA, 15260, USA

## Abstract

**Background:**

Septal cholinergic neurons account for most of the cholinergic innervations of the hippocampus, playing a key role in the regulation of hippocampal synaptic activity. Disruption of the septo-hippocampal pathway by an experimental transection of the fimbria-fornix drastically reduces the target-derived trophic support received by cholinergic septal neurons, mainly nerve growth factor (NGF) from the hippocampus. Axotomy of cholinergic neurons induces a reduction in the number of neurons positive for cholinergic markers in the medial septum. In several studies, the reduction of cholinergic markers has been interpreted as analogous to the neurodegeneration of cholinergic cells, ruling out the possibility that neurons lose their cholinergic phenotype without dying. Understanding the mechanism of cholinergic neurodegeneration after axotomy is relevant, since this paradigm has been extensively explored as an animal model of the cholinergic impairment observed in neuropathologies such as Alzheimer's disease.

The principal aim of this study was to evaluate, using modern quantitative confocal microscopy, neurodegenerative changes in septal cholinergic neurons after axotomy and to assess their response to delayed infusion of NGF in rats.

**Results:**

We found that there is a slow reduction of cholinergic cells labeled by ChAT and p75 after axotomy. However, this phenomenon is not accompanied by neurodegenerative changes or by a decrease in total neuronal number in the medial septum. Although the remaining axotomized-neurons appear healthy, they are unable to respond to delayed NGF infusion.

**Conclusions:**

Our results demonstrate that at 3 weeks, axotomized cholinergic neurons lose their cholinergic phenotype without dying and down-regulate their NGF-receptors, precluding the possibility of a response to NGF. Therefore, the physiological role of NGF in the adult septal cholinergic system is to support phenotypic differentiation and not survival of neurons. This evidence raises questions about the relationship between transcriptional regulation of the cholinergic phenotype by retrograde-derived trophic signaling and the transcriptional changes experienced when retrograde transport is impaired due to neuropathological conditions.

## Background

Basal forebrain cholinergic neurons (BFCN) account for most of the cholinergic innervation of the hippocampus and cortical mantle, and have a key role in the regulation of synaptic activity and modulation of memory and attention in rodents, primates and humans [1-5].

The physiology of septal cholinergic neurons is regulated by the trophic support offered by their target, the hippocampus. Disconnection of septal cholinergic neurons from their target by an experimental transection of the fimbria-fornix, reduces the number of neurons positive for cholinergic markers such as choline acetyl transferase (ChAT) [1,6-13]. One of the best-studied trophic factors for septal cholinergic neurons is nerve growth factor (NGF). Levels of NGF mRNA and protein are consistently detected in the hippocampal formation and cortex. In addition, it is well established that NGF is retrogradely transported from the hippocampus to the septal area and that intracerebroventricular application of NGF or intracerebral transplant of NGF-releasing cells prevents the reduction in the proportion of neurons positive for cholinergic markers after axotomy [14,7,12-18].

The mechanisms of cholinergic neurodegeneration after axotomy are poorly characterized. Most of the studies performed have considered the loss of ChAT, acetyl cholinesterase (AChE) or p75 neurotrophin receptor (p75) immunoreactivity as an analogue of neurodegeneration [19-22], ruling out the possibility that neurons lose their cholinergic phenotype without dying.

Septal cholinergic neurons express two different types of receptors for NGF: the tyrosine kinase receptor TrkA (TrkA), which specifically binds NGF; and the p75 neurotrophin receptor (p75), which binds all neurotrophins. Together, TrkA and p75 activate pro-survival gene expression and influence growth [23]. Conversely, p75 signaling mediates neuronal death by apoptosis in different neuronal systems, including neurodegenerative models such as corticospinal axotomy and seizure-induced apoptosis of septal cholinergic neurons [24-29]. Activation of NGF receptors up-regulates several cholinergic-specific genes, such as the high-affinity choline transporter and the acetylcholine synthesizing enzyme ChAT, which share a common gene locus with the vesicular acetylcholine transporter (VAChT) [30-33].

Axotomy-induced cholinergic decay in the basal forebrain has been explored as an animal model for cognitive decline due to cholinergic impairment, similar to that observed in aging and neuropathologies such as Alzheimer's disease (AD) [34,35]. Due to its role as a neurotrophic factor for cholinergic neurons, NGF gene therapy is currently in phase 1 clinical trial for AD treatment [12,36-39].

The principal aim of this research was to re-evaluate, using modern quantitative confocal microscopy, neurodegenerative changes in septal cholinergic neurons after axotomy and to assess their response to delayed infusion of NGF in rats. To pursue this goal, we have performed a stereological analysis of the rat septal area, using quantitative double- and triple-labeling confocal microscopy analysis of axotomized brains with different cell markers and neurodegenerative labels at different time-points after axotomy. Furthermore, we have assessed the response of cholinergic neurons to delayed infusion of NGF three weeks after axotomy.

As shown before, we found that there is a slow reduction in the number of immunoreactive cholinergic cells after axotomy [6,40]. However, this phenomenon is not accompanied by neurodegenerative changes or a decrease of total neuronal number in the medial septum. Although the remaining axotomized-cells appear healthy, they are unable to respond to delayed NGF infusion. These results demonstrate that axotomized cholinergic neurons down-regulate their NGF receptors, precluding the possibility of a response to NGF.

Our results suggest that the physiological role of NGF in the adult septal cholinergic system is to support phenotypic differentiation and not neuron survival. This evidence raises the question of how the connection with the target regulates the transcription of cholinergic markers *in vivo *and of which other factors could re-induce the cholinergic phenotype when retrograde transport is impaired due to neuropathological conditions such as AD and Down syndrome [38,41,42].

## Results

### Expression profile of ChAT and p75 immunopositive septal cholinergic neurons after axotomy of the fimbria-fornix: a time course study

**Figure 1:**
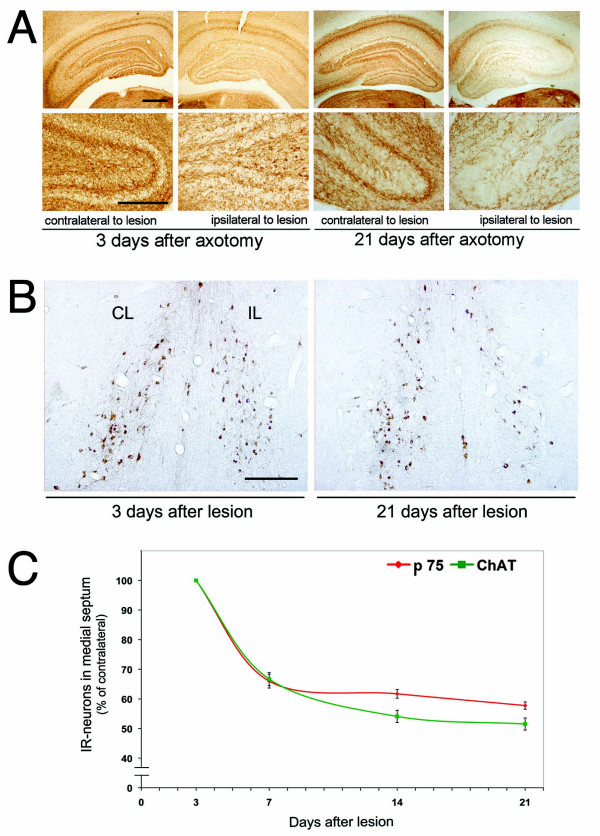
**Effect of septo-hippocampal pathway axotomy in cholinergic septal neurons**. **A**, Light microscopy of coronal sections stained for AChE 3 or 21 days after lesion procedure, showing the early effect of axotomy in cholinergic projections towards the hippocampus. Twenty-one days after axotomy, AChE-positive terminals are strongly reduced in the ipsilateral side of the lesion, as compared to the contralateral side (scale bar 1 mm). Inferior panels, magnification inset of AChE-positive neurites in dentate gyrus (scale bar 200 μm). **B**, Light microscopy of immunohistochemistry anti-ChAT in coronal sections, showing the effects of the axotomy in cell bodies of cholinergic septal neurons, 3 or 21 days after axotomy. Twenty-one days after the lesion, the number of ChAT-positive neurons is clearly reduced on the ipsilateral side of the medial septum (CL, contralateral; IL, ipsilateral. Scale bar: 1 mm). **C**, Time course of ChAT- and p75-positive neuron loss after axotomy. The graph shows the quantification of ChAT- or p75-immunopositive neurons in the medial septum from serial sections of brains at 3, 7, 14 and 21 days after the lesion. Note that the numbers of ChAT- and p75-immunopositive cells decay with similar kinetics but with a different slope between 7-14 days.

It is well established that after two weeks of unilateral axotomy of the fimbria-fornix, the number of ChAT-positive neurons ipsilateral to the lesion decreased to about 30% as compared to the contralateral side [8,10,13,43]. To quantify cholinergic cell loss, we used two different antibodies against well-established cholinergic markers, ChAT and p75. We found a similar slow reduction in ChAT and p75 immunopositive cells as well as diminished acetyl cholinesterase (AChE) fiber staining in the hippocampus (Figure 1). We observed that the number of cells labeled with ChAT or p75 decreased with similar kinetics over time (7 to 21 days after axotomy). However, there were always more cells labeled with p75, suggesting that some cholinergic cells had lost ChAT expression but continued expressing p75 (Figure 1C).

**Figure 2:**
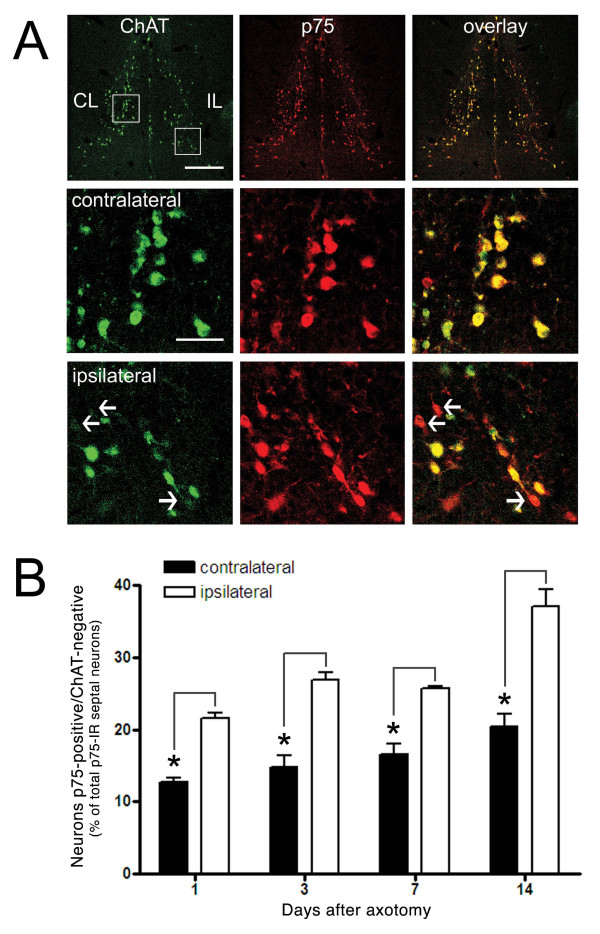
**The number of neurons that are positive only for p75 is increased in the ipsilateral side after axotomy**. **A**, Confocal microscopy of double-immunofluorescence anti-ChAT/anti-p75 reveals an increase in the number of neurons that are positive only for p75 at different times after the axotomy. The superior panel is a panoramic view of the medial septum at 14 days after axotomy (scale bar: 1 mm). The central and inferior panels are magnification insets (scale bar: 200 μm) of neurons contralateral and ipsilateral to the lesioned side, showing p75-positive and ChAT-negative cells as indicated by the white arrows. **B**, The graph shows the percentage of total septal neurons expressing p75 that are p75-positive and ChAT-negative at 1, 3, 7 or 14 days after axotomy. Black bars represent the control side of the septum and white bars represent the side ipsilateral to the lesion. Asterisk indicates significance level p < 0.05.

**Figure 3:**
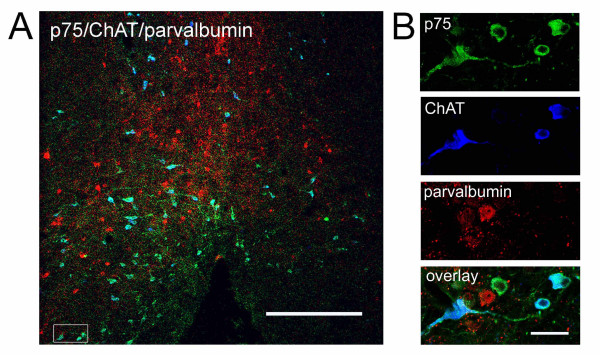
**p75-positive and ChAT-negative neurons are not GABAergic**. Confocal microscopy of triple-immunofluorescence anti-ChAT/anti-p75/anti-parvalbumin shows no significant colocalization of GABAergic marker and p75. Left, a panoramic view of the medial septum (scale bar: 1 mm) and panel showing a magnification inset of a representative group of neurons from triple-labeled sections (scale bar: 300 μm).

This finding was supported by quantitative confocal studies where both ChAT and p75 were labeled in the same section and the number of cells that were co-labeled with both proteins was quantified. On the non-axotomized side, co-localization between the two labels was about 90%, whereas 14 days after the lesion, just 60% of the neurons labeled with p75 were also positive for ChAT (Figure 2). In the septal nucleus, there are also GABAergic neurons that could be re-expressing p75 due to the axotomy. In a triple immunofluorescence with ChAT, p75 and parvalbumin 'a commonly used GABAergic marker [44,45]- there was a non-significant number of cells co-labeled with parvalbumin and p75 (Figure 3), ruling out the possibility that the neurons expressing just p75 are GABAergic.

### Septal cholinergic neurons do not degenerate through apoptotic cell death after axotomy

**Figure 4:**
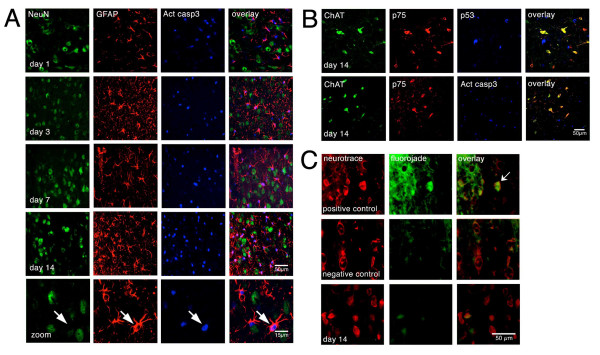
**Cholinergic septal neurons do not undergo apoptosis after axotomy**. **A**, Triple-immunofluorescence against Neu-N (neuronal marker), GFAP (astroglial marker) and activated Caspase-3 shows no colocalization of the apoptotic marker with neurons at different time points (1, 3, 7, 14 days) after axotomy. There is an increase in the number of cells that are immunopositive for activated caspase-3 with time. The correlation between GFAP and activated Caspase-3 suggests that astrocytes are undergoing apoptosis (scale bar: 50 μm). **B**, Triple-immunofluorescence against ChAT, p75 and p53, shows no colocalization of p53 (an early apoptotic marker) with p75- or ChAT-immunopositive neurons in the brains of axotomized rats 14 days after the axotomy. Confocal microscopy (scale bar: 50 μm). Triple-immunofluorescence against ChAT, p75 and activated caspase-3 shows no colocalization of activated Caspase-3 and p75 or ChAT immunopositive neurons after 14 days of axotomy. Confocal microscopy (scale bar 50 μm). **C**, Axotomized septal neurons are not labeled with Fluorojade C (a specific staining for degenerating neurons). Superior panel, a brain section from a rat injected in medial septum with 100 mM H_2_O_2 _was stained with Neurotrace and Fluorojade C as a positive control. The arrow indicates a degenerating neuron. Center panel, double-labeling with Neurotrace and Fluorojade in a brain section from an untreated rat. Inferior panel shows no colocalization of Neurotrace and Fluorojade C in medial septal neurons 14 days after axotomy (scale bar 50 μm).

p75 is an apoptotic receptor that mediates cell death of cholinergic neurons in other paradigms [27,46]. However, cholinergic neurons from p75 knockout mice are not protected from the reduction in numbers of cholinergic neurons after axotomy [19], suggesting that p75 does not play a role in cholinergic neurodegeneration after axotomy or that there is no death of cholinergic cells after the fimbria-fornix lesion. To further support this possibility, we performed triple immunostaining against ChAT, p75 and p53 or cleaved caspase-3 in rat brain sections starting from day one up to two weeks after axotomy. Although there were positive reactions for cleaved caspase-3 or p53 in the septal nucleus after axotomy, they did not co-localize with ChAT, p75 or the general neuronal marker NeuN (Figure 4A, B, C). In addition, neurons were not labeled with Fluorojade C, a well-known marker of degenerating neurons [27,47] (Figure 4D). To further identify the nature of the cells that were positive for cleaved caspase-3 (Figure 4C), double immunostaining was performed with NeuN [48,49] and the glial fibrillary acidic protein (GFAP) to label astroglia. We could clearly identify the astroglia as the cell type co-localizing with cleaved caspase-3 (Figure 4C).

To standardize the immunostaining of cleaved caspase-3 and p53, we induced cell death by injecting H_2_O_2 _to the medial septum. In these conditions, cleaved caspase-3 and p53 clearly labeled damaged neurons as indicated by their co-localization with altered Neurotrace labeling (Additional file 1). Neurotrace is a fluorescent Nissl staining that only labels neurons and does not co-localize with markers of different glial cells such as astro, micro or oligodendroglia (Additional file 2).

### There is no loss of neurons in the medial septum three weeks after axotomy

**Figure 5:**
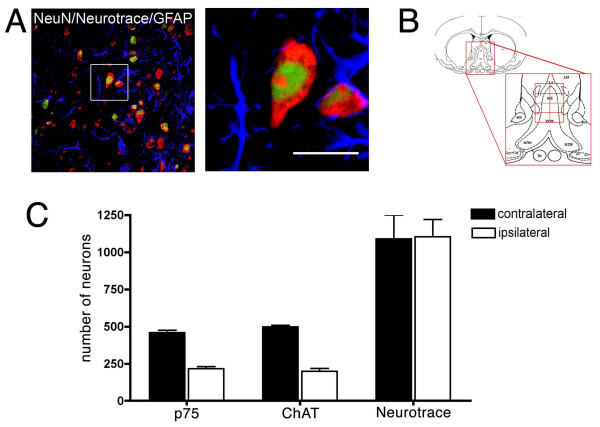
**There is no difference between the number of septal neurons when comparing the ipsilateral and contralateral regions of brains 21 days after axotomy**. **A**, Left, confocal microscopy of triple labeling for Neurotrace (red), NeuN (green) and GFAP (blue) from the septal region of a lesioned brain. In order to assure that we were counting only neurons, we used two neuronal markers and an astrocytic marker (GFAP). Right, inset magnification of a neuron (scale bar: 80 μm) showing the Neurotrace and NeuN labeling profiles. **B**, Diagram illustrating the area of the medial septum (MS) that was considered for the quantification of cholinergic or total number of neurons (adapted from Rat Brain Atlas [65]). Each side of the total area was divided to 4 fields and then photographed and manually quantified. The anatomical landmarks used to define the MS are also indicated (see Experimental Methods). cc, corpus callosum; LV, lateral ventricle; aca, anterior commissure. **C**, Quantification of p75-positive, ChAT-positive and total septal neurons. Comparison of the number of septal neurons on contralateral and ipsilateral sides shows differences in the numbers of p75- and ChAT-immunopositive neurons, but no significant difference in total number of neurons (n = 5; Student's t-test, p > 0.001, ± SD) 21 days after axotomy.

In order to determine whether there is a non-apoptotic neuronal degeneration after axotomy in the septal nucleus, triple immunostaining with two neuronal markers -Neurotrace and NeuN- plus the astroglial marker GFAP was performed in sections of 3-week axotomized rat brains and control brains. The total number of neurons in the septal nucleus was quantified as defined in Figure 5. Our analysis indicated that there was no difference between the total numbers of neurons from the contralateral vs. ipslateral side to the lesion (Figure 5). It is still possible that the axotomy performed to the rat brain may have an effect in the total number of neurons in the contralateral side to the lesion. To address this point, we have quantified the total number of neurons in the medial septum -as define in Figure 5- in a healthy brain comparing the left and the right sides of the medial septum. We found, no differences in the total number of neurons between the left and the right sides of healthy brains nor differences between these numbers and the numbers of neurons found in lesioned brains (Table 1). These results, together with the lack of cleaved caspase-3, p53 and Fluorojade C staining 3 weeks after axotomy, strongly support the notion that axotomy of the fimbria-fornix does not induce neuronal death in the medial septum. Our results are also consistent with the study of van der Zee and Hagg in p75 knockout (KO) mice. The authors studied the potential participation of p75 in the death of axotomized cholinergic cells [19], and found that the absence of p75 does not prevent septal ChAT-positive cell loss. However, these results should be taken with caution, since KO mice still express an alternatively spliced form of p75 [50].

**Table 1:** Total number of neurons in the medial septum.

	Number of neurons			
**Brain Hemisphere**	**Left (± SD)**	**Right (± SD)**	**Number of brains considered**	**Number of sections per brain**

Lesioned brain	1090 ± 319 (CL)	1103 ± 233 (IPL)	5	3

Healthy brain	1085 ± 137	1057 ± 82	3	3

Neurons from the medial septum, as delineated in Figure 5, of brains from axotomized rats (lesioned brains) and control rats (healthy brains) were labeled with neurotrace and their number quantified. CL, contralateral to the lesion. IPL, ipsilateral to the lesion.

### Response of axotomized septal neurons to delayed NGF infusion

**Figure 6:**
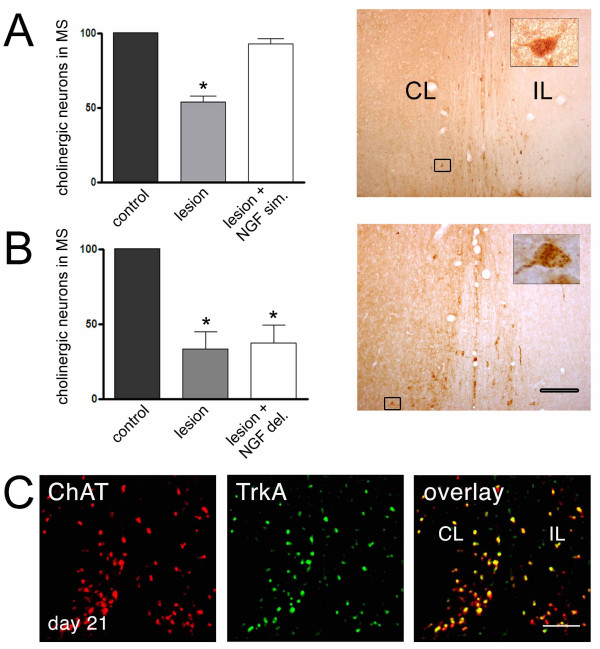
**Effect of intracerebroventricular infusion of NGF on the loss and recovery of the cholinergic phenotype**. **A**, The septohippocampal path was axotomized and NGF-infusion was performed for two weeks. Left, quantification of septal cholinergic neurons in the lesioned side as compared to the contralateral side in untreated animals (control), lesioned animals (lesion) and lesioned animals brain-infused with NGF (lesion + NGF sim.). Right, light microscopy of anti-NGF immunohistochemistry showing the wide distribution of infused NGF and uptake of NGF by septal neurons. Exogenous NGF infused right after axotomy protects cholinergic cells from ChAT-loss. **B**, The septohippocampal path was axotomized and 3 weeks after the axotomy (delayed NGF-infusion), NGF was infused for two additional weeks. This procedure did not protect cholinergic cells from ChAT-loss, contrary to the protection observed with simultaneous infusion. **C**, Confocal microscopy of double immunofluorescence against TrkA and ChAT showing the colocalization of these markers in septal neurons 21 days after axotomy (inferior panel). There are no ChAT-negative cells positive for TrkA, suggesting that neurons cannot respond to NGF 3 weeks post-axotomy due to down-regulation of NGF receptors.

It is possible that the neurons that survive after axotomy are still able to re-express the cholinergic markers if trophic support is restored. Normally, axotomized cholinergic cells respond to NGF when the infusion of NGF is concurrent with lesion surgery, as shown in Figure 6A and described previously [6,20,40]. However, when NGF was infused 3 weeks post-axotomy, neurons did not respond and there were no increase in cholinergic cell number, as is observed when NGF is infused simultaneously to the lesion (Figure 6B). This correlates with the fact that there is a reduction in the number of TrkA-positive cells on the ipsilateral side, and all remaining TrkA-positive cells are also ChAT positive (Figure 6C). This suggests that the lack of response to NGF is due to down-regulation of TrkA. The effect is not explained by an incomplete diffusion of NGF to the ipsilateral side of the septum, since there are neurons that are clearly positive for NGF: they show a punctate pattern of staining, which is consistent with receptor-mediated NGF uptake (Figure 6A, B). These results are apparently different from those reported by Hagg and colleagues [19,40] in mice. They found a small recovery of ChAT immunopositive cells in the ipsilateral side after delayed NGF treatment, in comparison to untreated animals. However, in agreement with our findings, van der Zee and Hagg observed that a significant number of neurons (60%) were not capable of responding to a delayed NGF infusion paradigm.

## Discussion

In the present study, we report the response of basal forebrain cholinergic cells to axotomy based on the use of time-course analyses, multiple immunostainings and quantitative confocal microscopy. Our results establish that cholinergic cells do not respond to disconnection of their target, as induced by axotomy, with apoptotic cell-death, but rather with down-regulation of the neurotransmitter-synthesizing enzyme ChAT and NGF receptors. As opposed to other nervous system injury models, such as corticospinal and olfactory bulb axotomy and kainic acid-induced cytotoxicity [27,29,51], p75 receptor does not play a role in axotomy-induced cholinergic cell neurodegeneration. Of note, like septal cholinergic cells, Purkinje cells in the cerebellum are also resistant to axotomy [52]. We are yet far from explaining the differences in the response to axotomy between cholinergic and Purkinje neurons and cortico-spinal and olfactory bulb neurons. One possibility is that cholinergic neurons became resistant to the withdrawal of neurotrophic factor, as postulated by Snider [53] for sensory neurons. It is well established that during development, or in neonates, axotomy or neurotrophic deprivation induces apoptotic cell death of dorsal root ganglia (DRG) neurons [54]. However, adult DRGs 'as opposed to embryonic DRGs- differentially express DeltaNp73a, a pro-survival protein related to the p53 family [55]. The expression of this protein renders cells resistant to axotomy or neurotrophic factor withdrawal. Another gene involved in protection of neurons after injury is the heat shock protein Hsp27, which directly or indirectly activates the Akt survival pathway. The expression of Hsp27 inhibits JNK-mediated apoptosis in superior cervical ganglion neurons and adult DRGs [56,57]. It would be interesting to study whether adult cholinergic cells express DeltaNp73 and/or Hsp27. As mentioned above, other adult neurons such as corticospinal neurons are not resistant to axotomy and die in a p75-dependent fashion, probably because they fail to express any of the abovementioned survival programs after injury [29,58].

One unexpected observation of our study was that only GFAP-positive cells were immunopositive for cleaved caspase-3. GFAP positive cells appear hypertrophic and strongly stained in the whole tissue, thus revealing a generalized astrogliosis. This reaction may account for a global inflammatory response to the lesion which may cause apoptosis of astrocytes, as has been reported in other injury models or in neurodegenerative diseases [59]. It is interesting that apoptotic cell death in the septal nucleus after axotomy was previously reported by electron microscopy studies [21]. However, the quality of the microscopy precludes discrimination of the cell type. Therefore, it is possible that the apoptotic septal cells mentioned in this study are glial cells rather than neurons.

Disruption of the connection between septal cholinergic cells and the hippocampus results in down-regulation of ChAT, but not neuronal death. A consequence of this is that the basal forebrain cholinergic cells affected during aging and in pathological states such as AD may still be alive. Therefore, the possibility of restoring the cholinergic phenotype by identifying new factors that influence cholinergic function would be an interesting point for further investigation. Some candidates are the BMPs and neurosteroids. Studies by Lopez-Coviella and colleagues have shown that BMP-9 is a robust factor for induction and maintenance of the cholinergic phenotype *in vitro *[60,61], which also synergizes with NGF to enhance neuronal transcriptional response [30,62]. In addition, neurosteroids such as estrogen and retinoic acid have also been shown to up-regulate the cholinergic phenotype in the septal basal forebrain [63,64].

Taking this into account, future studies could search for components that up-regulate the cholinergic phenotype in the absence of NGF receptors. This may open new avenues for therapeutic intervention for the treatment of the cholinergic deficit observed in AD.

## Methods

### Animals

Male Sprague-Dawley rats were maintained with free access to water and fed with normal rat chow at the Pontificia Universidad Catolica animal care facilities. Experimental procedures were in accordance with institutional standards for care and use of laboratory animals.

Rats weighting 280-300 g were anesthetized (xylazine 2 mg/ketamine 20 mg i.p. and lidocaine 9% locally applied on the ears) and positioned in a stereotaxic apparatus. Coordinates were calculated based on the Paxinos and Watson atlas of the rat brain [65]. After surgery, the animals were injected i.p with antibiotic (enrofloxacine 7.5 mg) and maintained under observation and temperature control for one hour. For histological preparation of brain tissue, rats were transcardially perfused with 250 ml of 0.9% NaCl, and 250 ml of 4% paraformaldehyde (PFA) in phosphate buffer. After extraction, the brain was post-fixed overnight in 4% PFA, left on 30% sucrose for 24 hours, and coronally sectioned (40 μm) on a cryostat.

### Fimbria-fornix transection

Unilateral axotomy of the septo-hippocampal pathway was induced by aspirative lesion of the fimbria-fornix, as has been previously described [66]. In brief, anesthetized rats were positioned in a stereotaxic apparatus and a small piece of skull was removed at the stereotaxic coordinates 1.8 mm caudal to the bregma, and 0.0-4.0 mm lateral to the midline. After excision of the dura, we performed a syringe aspiration of the dorsal fornix-fimbria. We also used a syringe to aspirate part of the cingulate and parietal cortices, 3.5 mm ventral from the brain surface. Rats were sacrificed 3-21 days after axotomy.

### H_2_O_2 _injection in the medial septum

Two μl of 0.1 M H_2_O_2 _were injected in the septal areas of two adult rats: 0.35 mm rostral to bregma, 0.5 mm lateral to midline and 7 mm dorso-ventral. After 72 hours, rats were perfused and brain sections were prepared for immunostaining.

### Intracerebroventricular infusion of NGF

Artificial cerebrospinal fluid (ACSF) containing 150 mM NaCl, 1.8 mM CaCl_2_, 1.2 mM MgSO_4_, 2 mM KH_2_PO_4 _and 10 mM glucose, pH 7.4 with or without NGF (Alomone Labs, Jerusalem, Israel) at a concentration of 0.2 μg/ml was infused for 14 days (2.5 μL/hour) by using a brain infusion kit (Alza Corp., Palo Alto, CA), connected to a model 2002 Alzet osmotic pump (Alza Corp., Palo Alto, CA), as described previously [6]. The cannulae and connector tube were filled with ACSF only or with ACSF plus NGF and attached to a loaded pump. Using the arm of the stereotaxic apparatus, the cannula was lowered into the brain at left ventricle coordinates (0.8 mm caudal to bregma, 1.2 mm lateral to midline and 3.5 mm ventral to the brain surface) and finally anchored to the skull with a screw and glued with dental acrylic. Axotomized rats were infused for 14 days immediately after axotomy (simultaneous NGF infusion) or 3 weeks after axotomy (delayed NGF infusion).

### AChE enzyme-histochemistry

Serial coronal cryostat sections (40 μm) were collected in 0.1 M phosphate buffer (pH 7.4), washed in 65 mM sodium maleate (pH 6.0) and incubated for staining, as floating sections, for 1 hour at room temperature in 0.05 mg/mL acetylthiocholine iodide, 0.1 tetra-isopropyl-pyrophosphatamide, 0.05 mM potassium ferricyanide, 0.3 mM CuSO_4_, 0.5 mM sodium citrate, and 65 mM sodium maleate (pH 6.0), as described previously [46].

### Immunohistochemistry

ChAT immunohistochemistry was performed as follows: (i) 15 min incubation in 0.03% H_2_O_2 _in 0.1 M Tris-HCl, 150 mM NaCl, pH 7.4 (TBS) to block endogenous peroxidase; (ii) 30 min incubation at 4°C with 0.4% Triton-X100 in TBS; (iii) 1.5 hr incubation at 4°C with 0.2% Triton-X100, 5% rabbit serum, 5% BSA in TBS; (iv) 48 hr incubation at 4°C with goat anti-ChAT antibody (Chemicon, CA, USA) diluted 1:300 in TBS plus 0.2% Triton-X100 and 5% serum; (v) 1.5 hr incubation at room temperature with biotin-conjugated rabbit anti-goat IgG (1:300 in TBS; DakoCytomation, CA, USA); (vi) 1 hr incubation with peroxidase-conjugated avidin ABC (DakoCytomation, CA, USA), followed by visualization of peroxidase activity with diaminobenzidine (DAB, 1 mg/mL) 0.01% H_2_O_2 _in TBS.

NGF immunohistochemistry was preceded by 15 min incubation in 50% ethanol, followed by the same protocol already described. Rabbit anti-NGF (Alomone Labs, Jerusalem, Israel) and rabbit anti-p75 (Upstate, NY, USA) were used at 1:300 and 1:500, respectively.

### Immunofluorescence

Single or double-immunofluorescence was performed in floating brain sections as follows: (i) 15 min incubation in TBS; (ii) 15 min incubation in NaBH_4 _10 mg/mL; (iii) 30 min incubation at 4°C with 0.4% Triton-X100 in TBS; (iv) 1.5 hr incubation at 4°C in 0.2% Triton-X100, 5% rabbit serum, 5% BSA in TBS; (v) 48 hr incubation at 4°C with primary antibodies in 0.2% Triton-X100 5% serum in TBS; (v) 1.5 hr incubation at room temperature with fluorochrome-conjugated (Molecular Probes, Oregon, USA) or biotin-conjugated secondary antibodies (directly labeled antibodies were used 1:100 in TBS; antibodies amplified with biotin were diluted 1:300 in TBS), followed by 1.5 hours with fluorochrome-conjugated streptavidin (Molecular Probes, Oregon, USA). Mouse anti-Neu-N, anti-parvalbumin, and rabbit anti-GFAP (labeling astroglia) antibodies were from Chemicon (Temecula, CA, USA). Mouse anti-CD11B (labeling microglia) was from Serotec, Oxford, UK. Rabbit polyclonal anti-OMgp (labeling oligodendroglia) was kindly provided by Dr. Colman (McGill University, Montreal, Canada). Polyclonal rabbit anti-cleaved caspase-3 was purchased from Cell Signaling Technology (Danvers, MA, USA). Monoclonal anti-p53 was from Santa Cruz Biotechnologies, CA, USA; polyclonal rabbit anti-TrkA antibody was kindly provided by Dr. L. Reichardt (University of California, San Francisco, CA, USA).

### Neurotrace and Fluorojade C staining

Staining with fluorescent probes such as Neurotrace (fluorescent Nissl stain; Molecular Probes, Oregon, USA) and Fluorojade C (specific marker for degenerating neurons; Chemicon, CA, USA) was performed as indicated by the manufacturers' instructions. Briefly, Neurotrace staining was performed after immunostaining by incubating brain sections for 20 min in a 1:200 dilution of Neurotrace in TBS. Sections were then rinsed, air-dried and mounted in Mowiol. Fluorojade staining was performed after Neurotrace as follows: brain sections were rinsed twice in TBS, re-hydrated for 2 minutes in distilled water and incubated for 10 minutes in 0.06% potassium permanganate. Finally, brain sections were washed for 2 minutes in distilled water and incubated for 10 minutes in 0.0002% Fluorojade C solution in 0.1% acetic acid. Sections were immediately rinsed in distilled water, mounted, air-dehydrated, cleared with xylene and mounted in Entellan.

### Septal cholinergic cell counting

The cholinergic cell count was performed essentially as described before [46] but modified for rat. Septal cholinergic neurons were defined by using anatomical landmarks in accordance with the rat brain atlas [65]. The ventral border of the medial septum was defined dorsal to the anterior commissure, and the rostral beginning was indicated by the meeting of the body of the corpus callosum at the midline. The caudal end of the septal nucleus was defined by the appearance of the fornix and the midline crossing of the anterior commissure. Four 40-μm-thick coronal sections were examined for each rat (n = 4 animals per time point), starting 0.7 mm caudal to bregma and 200 μm apart, to avoid counting the same cell twice. For each section, immunopositive neuronal profiles (labeled with ChAT or p75) were counted on images digitized on an Olympus BX51 (Tokyo, Japan) optic microscope (40× objective), equipped with a CoolSnap-Pro digital camera (Media Cybernetics, Maryland, USA) and connected to an image analysis system based on Image-pro express software, version 5.1.0.12 (Media Cybernetics, Maryland, USA). The pictures were analyzed by using the Sigmascan software (SPSS; Chicago, IL, USA). The criterion for identifying ChAT- or p75- immunopositive cells was the appearance of a clear nucleus or, in cases when staining was too dark, clear neuronal morphology.

### Total neuron counts

The anatomical landmark we used to define the medial septum nucleus (MS) was the same previously described for septal cholinergic cell counting. Three 40-μm-thick coronal sections were considered for each rat (n = 5 animals), starting 0.7 mm caudal to the bregma and 200 μm apart, to avoid counting the same cell twice. The sections were double-stained with Neurotrace (fluorescent Nissl stain; Molecular Probes, Oregon, USA; as indicated by the manufacturer instructions) and anti-Neu-N diluted 1:250 and developed with a secondary antibody conjugated to Alexa 488 fluorochrome. The area for counting was defined as a triangle: its base was a horizontal line crossing the middle point of the left and right anterior commissures, and its sides were the anatomical borders of the medial septum, as shown in Figure 5. Eight pictures of this area (four per side) were obtained using a confocal microscope with a 63× objective and scanned with an optical section of 10.3 μm. The criterion used to define healthy neurons was Neu-N and/or Neurotrace staining: cells with cytoplasmic and nucleolar staining, as shown in Figure 3, were counted as healthy neurons. Other patterns of staining corresponding to neurons undergoing degeneration [67,68], such as perinuclear Nissl (or chromatolytic) staining and eccentric distribution of the nucleus, were scarcely observed after axotomy (and clearly observed after H_2_O_2 _injection) and were not considered.

### Confocal microscopy

Confocal images for counting double-labeled ChAT/p75 neurons or total neurons labeled with Neurotrace and Neu-N were collected on a Zeiss LSM Pascal 5 (including a triple laser module [Arg 458/488/514 nm, HeNe 543 nm, HeNe 633 nm; Carl Zeiss, Thornwood, NY]) connected to an inverted microscope (Axiovert 2000). A lower objective (20×) was used to have a panoramic view of the septal nucleus of each brain section and a higher magnification objective (63×) was used to scan the total area as described in Figure 5. The images were analyzed using the SigmaScan software (SPSS, Chicago, IL, USA).

### Data analysis

Comparisons between the axotomized or unlesioned side of the septum were statistically validated by performing a Student's t-test to determine significance level (p < 0.05). The analyses were performed using the septum contralateral to the lesioned side as a control (100%).

## Competing interests

The authors declare that they have no competing interests.

## Authors' contributions

OML and JCM participated in design and performed all the experiments. CP participated in design and performed the analysis of the number of total neurons in healthy brains. OML and FCB drafted the manuscript. FCB conceived and coordinated the study and participated in design of all the experiments. NCI helped to coordinate the axotomy experiments and the manuscript drafting and critically reviewed the drafts. All authors read and approved the manuscript.

## Supplementary Material

Additional File 1 **Validation of p53 and activated-Caspase 3 as neurodegeneration markers in the septum**. Confocal microscopy of coronal sections triple-labeled with anti-activated caspase-3, anti-p53 and Neurotrace (fluorescent Nissl stain) was performed in septa of rats injected with H_2_O_2 _(100 mM) or unlesioned control rats. The unlesioned brain shows no reactivity for p53 or activated-caspase 3, while in the H_2_O_2_-injected brain there are labeled neurons in the region of injection. Labeling neurons (Neurotrace-positive cells) with anti-p53 and anti-activated-caspase 3, validates these antibodies as neurodegeneration markers. Additional file: descriptions text (including details of how to view the file, if it is in a non-standard format). Click here for file

Additional File 2 **Neurotrace is a specific neuronal marker**. Neurotrace is a specific neuronal stain and does not colocalize with glial markers. Left, confocal microscopy (scale bar: 500 μm) of sections double-labeled with Neurotrace and astroglia (top, anti-GFAP), microglia (center, anti-CD11B), oligodendroglia markers (bottom, anti-OMgp). Right panel shows inset magnifications (scale bar: 150 μm). Click here for file
