# Supplementary figures and images for: Axotomy-induced neurotrophic withdrawal causes the loss of phenotypic differentiation and downregulation of NGF signalling, but not death of septal cholinergic neurons
Source: Mol Neurodegener. 2010 Jan 19;5:5. doi: 10.1186/1750-1326-5-5 (PMC2826326; doi:10.1186/1750-1326-5-5)

Neurotrace

Act casp3

p53

overlay

unlesioned

H<sub>2</sub>O<sub>2</sub> injected

150μm

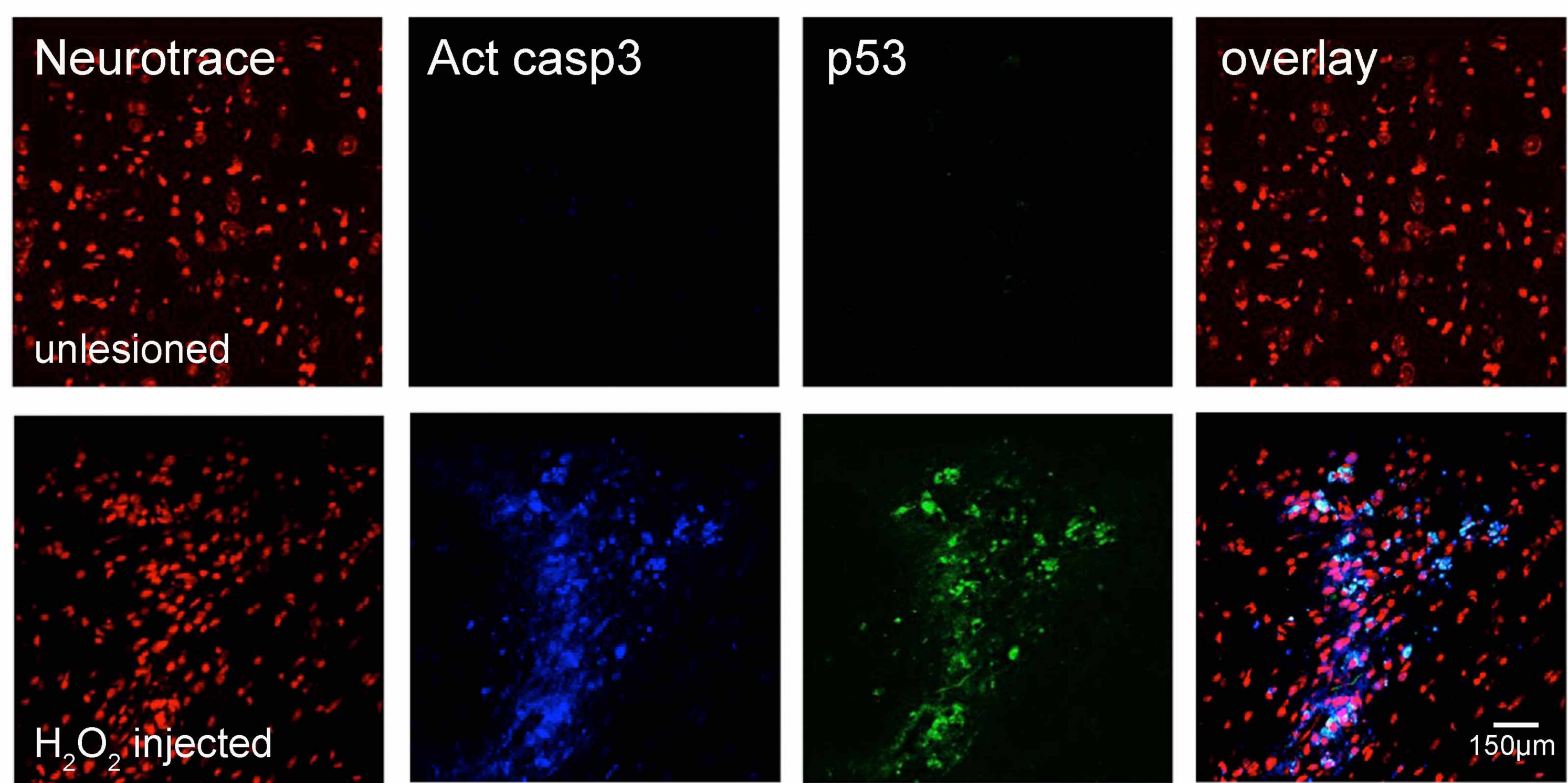

Supplement: Additional File 1 — Validation of p53 and activated-Caspase 3 as neurodegeneration markers in the septum. Confocal microscopy of coronal sections triple-labeled with anti-activated caspase-3, anti-p53 and Neurotrace (fluorescent Nissl stain) was performed in septa of rats injected with H2O2 (100 mM) or unlesioned control rats. The unlesioned brain shows no reactivity for p53 or activated-caspase 3, while in the H2O2-injected brain there are labeled neurons in the region of injection. Labeling neurons (Neurotrace-positive cells) with anti-p53 and anti-activated-caspase 3, validates these antibodies as neurodegeneration markers. Additional file: descriptions text (including details of how to view the file, if it is in a non-standard format). [file 1750-1326-5-5-S1.pdf]

neurotrace/astroglia

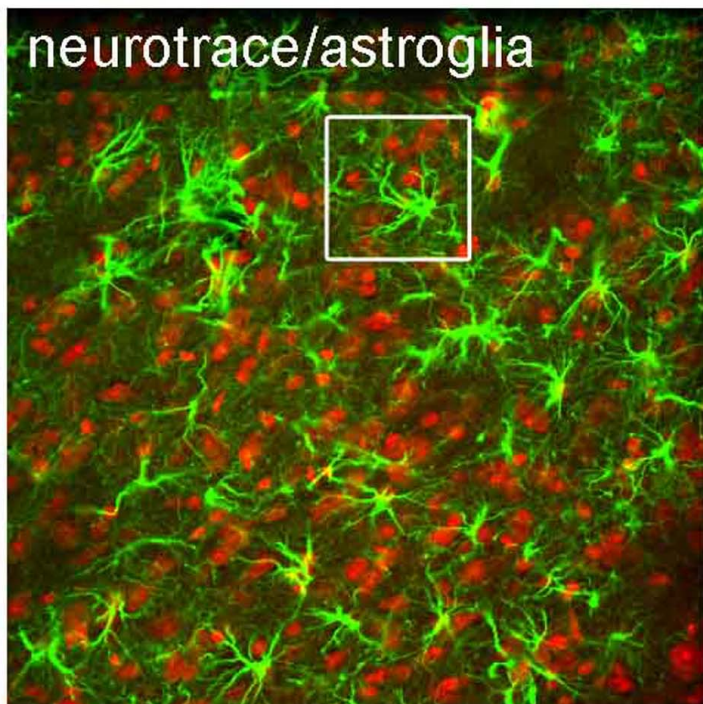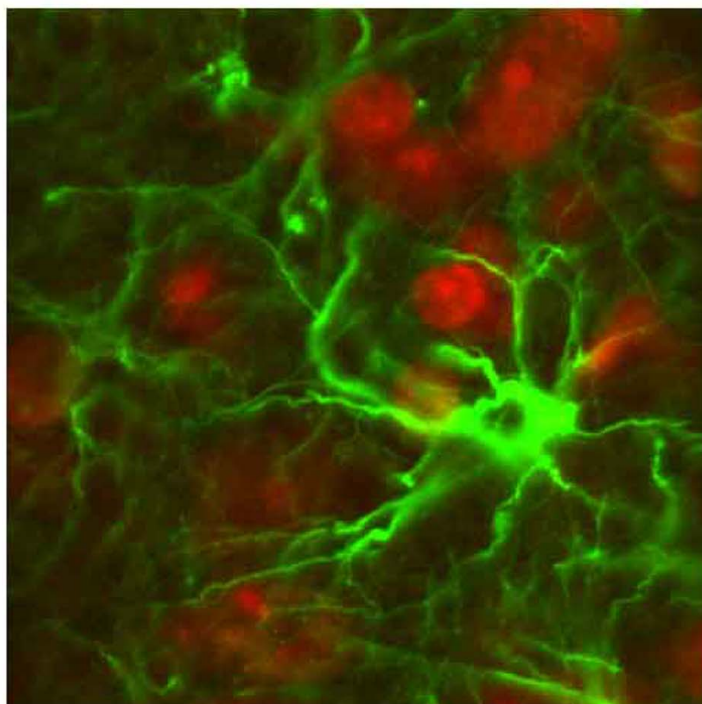

neurotrace/microglia

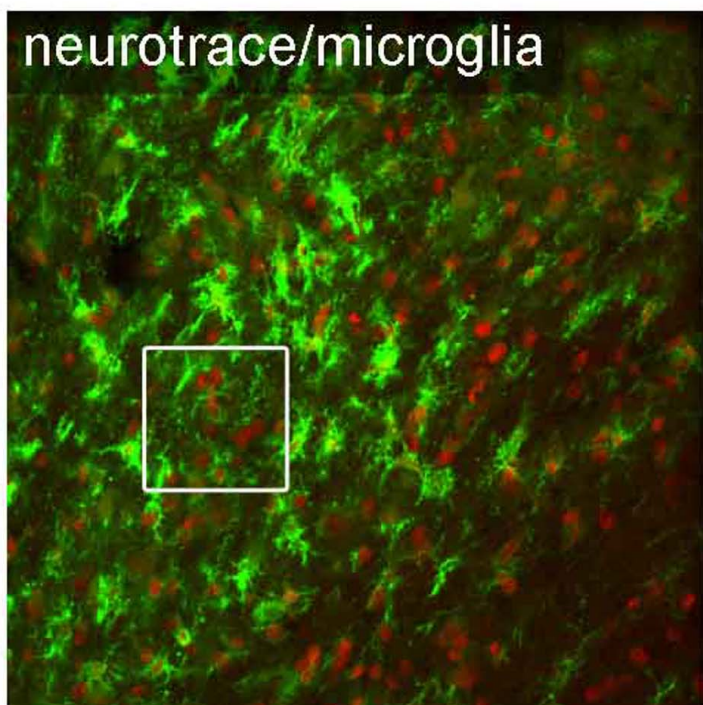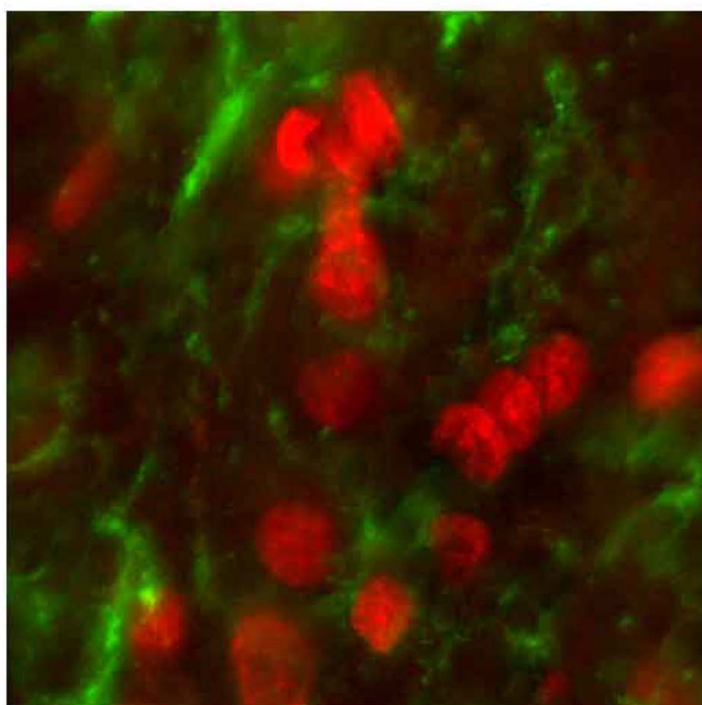

neurotrace/oligodendroglia

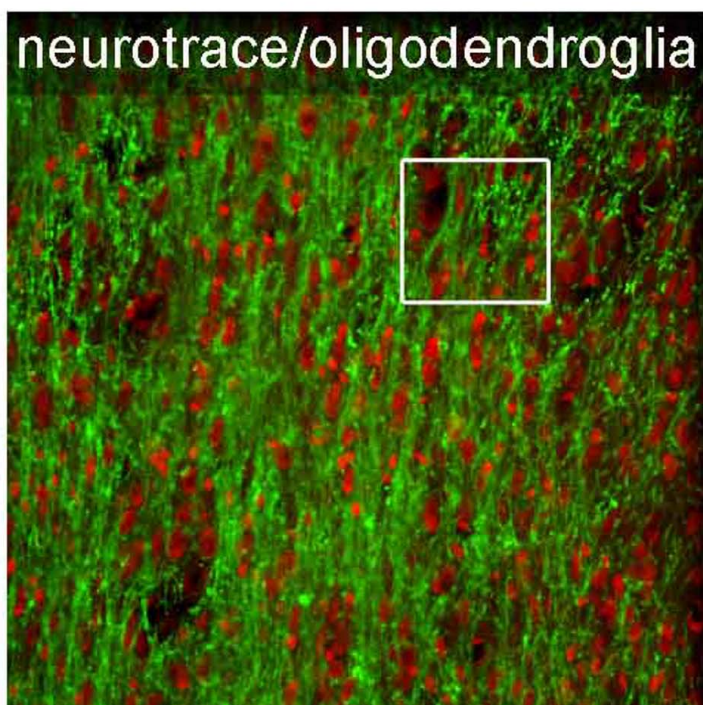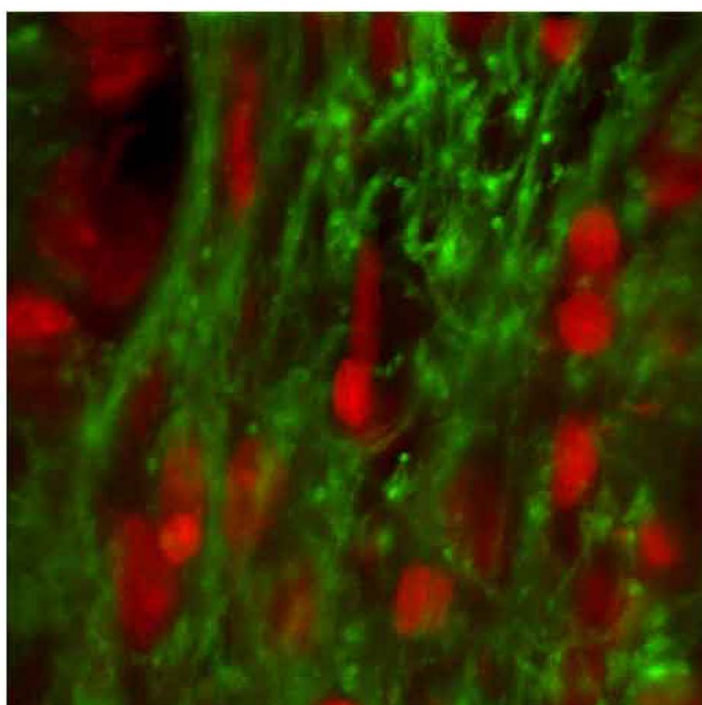

Supplement: Additional File 2 — Neurotrace is a specific neuronal marker. Neurotrace is a specific neuronal stain and does not colocalize with glial markers. Left, confocal microscopy (scale bar: 500 μm) of sections double-labeled with Neurotrace and astroglia (top, anti-GFAP), microglia (center, anti-CD11B), oligodendroglia markers (bottom, anti-OMgp). Right panel shows inset magnifications (scale bar: 150 μm). [file 1750-1326-5-5-S2.pdf]
